# Polyglycerol-Based Hydrogel as Versatile Support Matrix for 3D Multicellular Tumor Spheroid Formation

**DOI:** 10.3390/gels9120938

**Published:** 2023-11-29

**Authors:** Boonya Thongrom, Peng Tang, Smriti Arora, Rainer Haag

**Affiliations:** Institute for Chemistry and Biochemistry, Freie Universität Berlin, Takustr. 3, 14195 Berlin, Germanypeng.tang@fu-berlin.de (P.T.)

**Keywords:** polyglycerol-based hydrogel, thiol-Michael click reaction, stiffness, ECM-mimicking platform, 3D tumor spheroids

## Abstract

Hydrogel-based artificial scaffolds are essential for advancing cell culture models from 2D to 3D, enabling a more realistic representation of physiological conditions. These hydrogels can be customized through crosslinking to mimic the extracellular matrix. While the impact of extracellular matrix scaffolds on cell behavior is widely acknowledged, mechanosensing has become a crucial factor in regulating various cellular functions. cancer cells’ malignant properties depend on mechanical cues from their microenvironment, including factors like stiffness, shear stress, and pressure. Developing hydrogels capable of modulating stiffness holds great promise for better understanding cell behavior under distinct mechanical stress stimuli. In this study, we aim to 3D culture various cancer cell lines, including MCF-7, HT-29, HeLa, A549, BT-474, and SK-BR-3. We utilize a non-degradable hydrogel formed from alpha acrylate-functionalized dendritic polyglycerol (dPG) and thiol-functionalized 4-arm polyethylene glycol (PEG) via the thiol-Michael click reaction. Due to its high multivalent hydroxy groups and bioinert ether backbone, dPG polymer was an excellent alternative as a crosslinking hub and is highly compatible with living microorganisms. The rheological viscoelasticity of the hydrogels is tailored to achieve a mechanical stiffness of approximately 1 kPa, suitable for cell growth. Cancer cells are in situ encapsulated within these 3D network hydrogels and cultured with cell media. The grown tumor spheroids were characterized by fluorescence and confocal microscopies. The average grown size of all tumoroid types was ca. 150 µm after 25 days of incubation. Besides, the stability of a swollen gel remains constant after 2 months at physiological conditions, highlighting the nondegradable potential. The successful formation of multicellular tumor spheroids (MCTSs) for all cancer cell types demonstrates the versatility of our hydrogel platform in 3D cell growth.

## 1. Introduction

There is a strong emphasis on developing promising strategies to advance therapeutic approaches, including tissue engineering, regenerative medical treatments, and personalized therapies [1,2,3]. These strategies involve the extraction of a patient’s cells and their encapsulation within a three-dimensional scaffold. This scaffold serves as a temporary structural support for in vitro cell/tissue culture. Ideally, the scaffold’s design should closely mimic the composition, rigidity, and structure of the native tissue’s extracellular matrix or enable cells to remodel it, creating an environment conducive to cell function [4,5]. It is well recognized that the rigidity of the extracellular matrix plays a critical role in influencing cell behavior, such as spreading [6], migration [7], proliferation [8], and stem cell differentiation [9]. Thus, the development of a three-dimensional matrix with adjustable dynamic properties to replicate the temporal, structural, and mechanical aspects of extracellular matrix dynamics is of utmost importance in understanding its impact on cell behavior [10,11,12,13,14,15]. An oversimplified 2D model, which lacks tumor microenvironment and tumor heterogeneity, does not meet the requirements for advanced biological analysis. However, ethical problems evoked by animal experiments are not to be neglected. In order to bridge the gap between 2D cell cultures and in vivo models, it is necessary to establish multicellular tumor spheroids (MCTSs) models [16]. The traditional methodologies for tumoroid fabrication, e.g., hanging droplets and [17]. non-adherent plate cultures [18], can form large tumoroids relatively fast. However, these techniques are still limited in their applications, lacking further support for long time preservation. Employing hydrogels as a platform for 3D culture not only can offer cell suspension and nonadherent conditions for aggregation into multicellular tumor spheroids (MCTSs) but also allows cancer stem cells (CSCs) expansion in a controllable manner [19].

Hydrogels, particularly synthetic ones, have emerged as primary candidates for in vitro cell/tissue culture due to their ability to closely mimic native cellular environments, including matrix rigidity [20], and sequester proteins [21]. Native tissues exhibit a wide range of stiffness, from soft brain tissue (260–490 Pa) to stiffer bone tissue (2–4 GPa) [22]. The mechanical rigidity of the matrix is a critical factor in modulating cell interactions with the surrounding extracellular matrix (ECM) and thereby influences fundamental cellular processes. The choice of a hydrogel is contingent upon the specific experimental requirements, such as the needed stiffness, optical characteristics, and conductive properties. One example of a synthetic hydrogel is using crosslinked polymers where the crosslinking between individual polymer molecules maintains the overall 3D structure of the hydrogel after it swells in an aqueous medium. When used for 3D cell culture, it is essential that not only the polymer material but also the crosslinking reaction is compatible with cell viability. Furthermore, the hydrogel’s stiffness and the pore architecture can be tuned by using two main approaches: varying the concentration of crosslinkers in the pre-polymer solution and adjusting the degree of polymerization.

Generally, a synthetic polymeric gel precursor comprising several hydroxy groups, and a polyether backbone like polysaccharides, can be an excellent option for the fabrication of a hydrogel since it provides hydrophilicity toward water which is vital to all living beings and is inert to microorganisms [23,24]. Dendritic polyglycerol (dPG), a bioinert multivalent polyether polymer, can be a potential alternative gel precursor for the formationof a hydrogel network. With the benefit of a large number of multivalent hydroxy groups on the surface of dPG, any postpolymerization modifications of potential moieties including crosslinking functional groups can be made with ease. Furthermore, dPG serves as a biocompatible material and has been studied exclusively in the field of biomedical applications [25,26,27]. The polyether-polyol scaffold is therefore an ideal hub for fabricating a biocompatible hydrogel network.

The primary objective of this study is to uncover the distinct roles of matrix stiffness in regulating the 3D culture patterns of different cancer cell lines originating from different cancerous tissues. This investigation is conducted using a non-degradable hydrogel system based on cytocompatible bioinert dendritic polyglycerol (dPG) and 4-arm polyethylene glycol (4-arm PEG). These 2 combinations are excellent not only for building biocompatible hydrogels but also for cost-effective upscale synthesis based on thiol-click chemistry. Notably, key matrix properties such as swelling capacity and hydrolytic stability remain constant.

## 2. Results and Discussion

### 2.1. Synthesis of Gel Precursors and Hydrogel Formation

**Figure 1:**
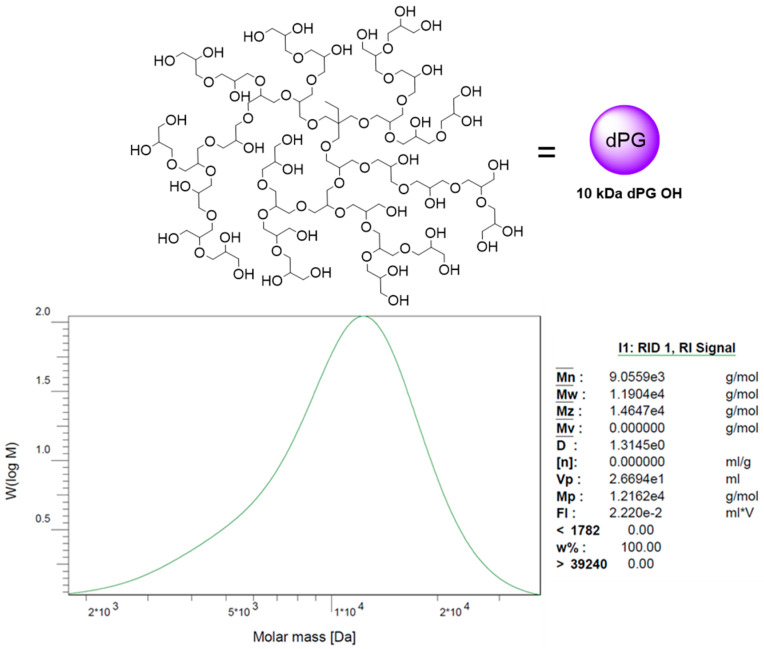
GPC chromatogram of 10 kDa dendritic polyglycerol (dPG) and illustrating picture of dPG structure.

The hydrogel was specifically designed to facilitate the growth of cancer cells, which are embedded in situ during the gelation process. The primary objective for this hydrogel is to maintain a stable and durable crosslinked structure that can securely hold the encapsulated cells within a 3D network for an extended period until a noticeable increase in their size is observed. The gel stiffness and consistency were influenced by the previous study from our group [28]. In the former study, we showed that cancer cell MCF7 could grow well in a 3D softer texture gel matrix whose shear storage modulus or stiffness is approximately in the range of 0.1–0.7 kPa and they successfully formed multicellular tumor spheroids (MCTSs) after the incubation for roughly 2 weeks. With this result, we have been inspired to further investigate the growth of other cancer cell lines by using the same hydrogel components which are dendritic polyglycerol (dPG) as a multivalency hub and 4-arm polyethylene glycol (4-arm PEG) as a crosslinker. The average size of the synthesized dPG polymer used in this study is approximately 10 kDa, based on a chromatogram result from Gel Permeation chromatography (GPC), and its hyperbranched architecture is demonstrated in Figure 1. However, the degradability has been modified in this study in order to prolong the stability of a hydrogel for intensive monitoring of MCTSs growth in a long incubation period.

**Figure 2:**
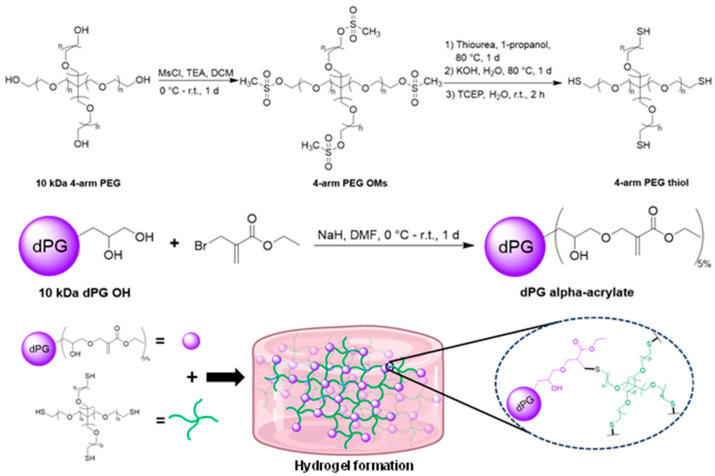
Synthetic routes of dPG alpha acrylate and 4-arm PEG thiol, and the schematics showing hydrogel formation.

dPG was designed to be functionalized with a nondegradable functional group which has similar reactivity as the acrylate group in the previous study. By using ethyl 2-(bromomethyl) acrylate as a reagent, the dPG alpha-acrylate was synthesized (Figure 2). This alpha-acrylate moiety serves as a nondegradable part of a hydrogel, with only ether and thioether bonds forming after the synthesis and gelation respectively [29]. The number of alpha-acrylate moieties decorated on the dPG was approximately 7 groups (the calculation can be seen in Supplementary Information). On the other hand, the crosslinker 4-arm PEG thiol was synthesized following the previous reports [30,31] with slight modification, thereby obtaining 4-arm PEG thiol with approximately 3.5 thiol groups, determined from Ellman’s assay (Figure 2).

The hydrogel is formed through a Michael addition reaction between the alpha-acrylate part and the thiol part by combining both gel components, including dPG alpha-acrylate and 4-arm PEG thiol, in either an aqueous solution or cell culture medium, as depicted in Figure 2. In the case of the encapsulation of cancer cells, both gel components and a culture medium solution containing the cancer cells were prepared and simultaneously loaded into a well within a 48-well plate (refer to Supplementary Information for specific details). After forming the hydrogel, it was allowed to set for approximately 4 h before the addition of culture medium for the subsequent cell growth experiment.

### 2.2. Characterizations of Hydrogel

**Figure 3:**
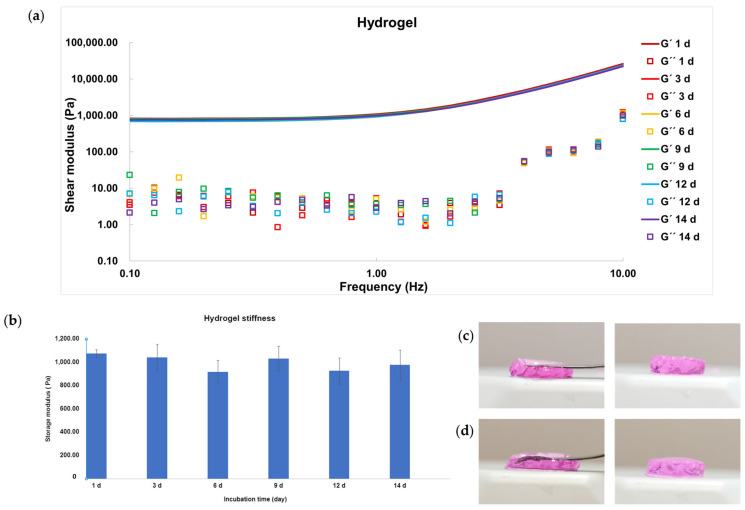
Hydrogel characterization: (**a**) Shear modulus graph performed by frequency sweep test at 37 °C of hydrogel sample at different incubation times; (**b**) Stiffness bar chart obtained from G′ value at 1 Hz from frequency sweep test of hydrogel sample at each incubation time; (**c**,**d**) Photographs of hydrogel prepared by using culture medium at 1 day and 14 days of incubation, respectively.

As mentioned in the earlier study, the optimal storage modulus or stiffness for the ideal 3D matrix consistency for cancer cell growth falls within the range of 0.1–0.7 kPa. Therefore, we fine-tuned the hydrogel stiffness and determined that at a gel concentration of 3% *w*/*v* and a mole ratio of 3:1 between dPG alpha-acrylate and 4-arm PEG dithiol, the gel exhibited an approximate stiffness of 1 kPa, showing soft texture (Figure 3c,d).

**Figure 4:**
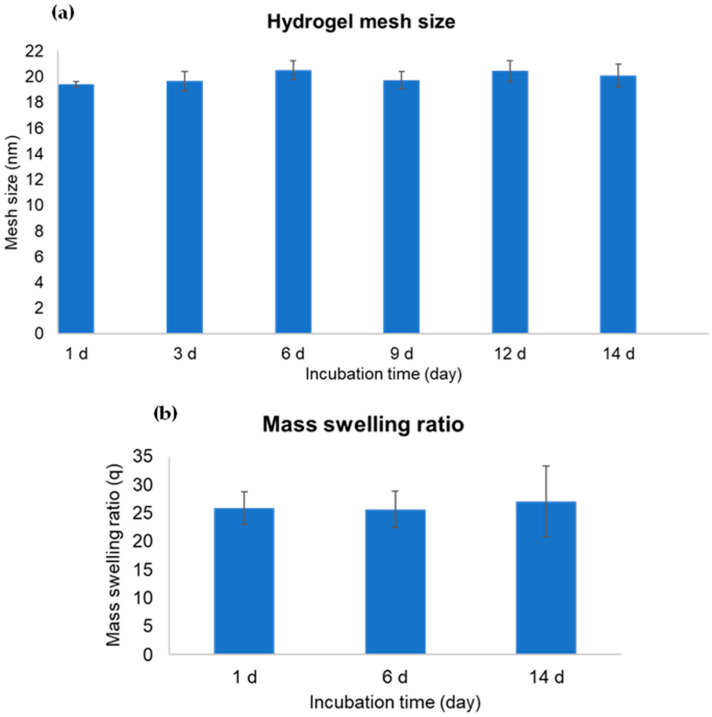
Hydrogel characterization: (**a**) Mesh size bar chart calculated from the stiffness value of the gel sample at each incubation time; (**b**) Mass swelling ratio of hydrogel sample at different incubation times.

The hydrogel created with dPG alpha-acrylate and 4-arm PEG thiol is typically nondegradable, primarily because of its ether and thioether linkages. This is in contrast to our earlier studies, where we employed degradable hydrogels linked by acrylate groups containing degradable ester bonds [28,30]. To prove the degradation concept, we, therefore, performed the degradation study for 2 weeks by using the hydrogel candidate without encapsulating cells. The gel sample was incubated at 37 °C with an excess of culture medium and the gel was picked at each time point for the rheological characterization of a shear storage modulus performed by rheometer. The resulting graph of shear storage (G′) and loss (G″) moduli of the sample at different time points (1 d, 3 d, 6 d, 9 d, 12 d, and 14 d) is shown in Figure 3a. By analyzing the storage modulus (G′) which represents the solid behavior or energy storage inside the chemically crosslinked networks, it is obvious that there is no difference between the G′ of 1 d, 6 d, and 14 d gel incubation. In addition, we can simply investigate the stiffness which represents a G′ value picked at 1 Hz, and the mesh size which was calculated from the stiffness value. The stiffness bar chart shows that sample at different incubation times has quite comparable stiffness value at approximately 0.9–1 kPa (Figure 3b). Similarly, the hydrogel mesh size of each time point also shows the same value close to 20 nm (Figure 4a). These results, hence, support that the hydrogel is unable to degrade in a cell culture medium at physiological pH and temperature for at least 2 weeks of incubation.

Besides the rheological data, the hydrogel sample was characterized by a mass swelling ratio. This mass swelling ratio can be calculated by dividing the mass of a swollen gel by the mass of a dried gel [32]. The hydrogel samples at day 1, day 6, and day 14 of incubation were chosen and the resulting mass swelling ratio can be seen in Figure 4b. From the bar chart, the gel samples at all 3 different time points show a high-water swelling capacity up to approximately 25 times from the dried state. The viscosity data obtained from a viscosity test with shear rate ramping from 0.1 to 10 s^−^^1^ in 2 min of the sample at different incubation times was also present (Figure S6). The data show the shear thinning behavior of the hydrogel, meaning that it has a lower viscosity at a higher shear rate. Based on the findings related to mass swelling ratio, shear stiffness, and viscosity, we anticipate that the sample should possess a soft texture with excellent network flexibility. We aimed to have this hydrogel effectively serve as a supportive ECM-mimicking platform for the formation of 3D multicellular tumor structures.

### 2.3. Formation of 3D Multicellular Tumor Spheroids (MCTSs)

To closely mimic the mechanical properties of extracellular matrix (ECM) for studying cell-environment interactions in ex vivo models, hydrogels emerge as an excellent choice due to their straightforward tunability [26]. Acrylate-based hydrogels are notable for their impressive biocompatibility and intriguing mechanical attributes [28,30,33]. Therefore, a meticulously engineered acrylate-based hydrogel platform greatly enhances the feasibility of various cellular investigations.

**Figure 5:**
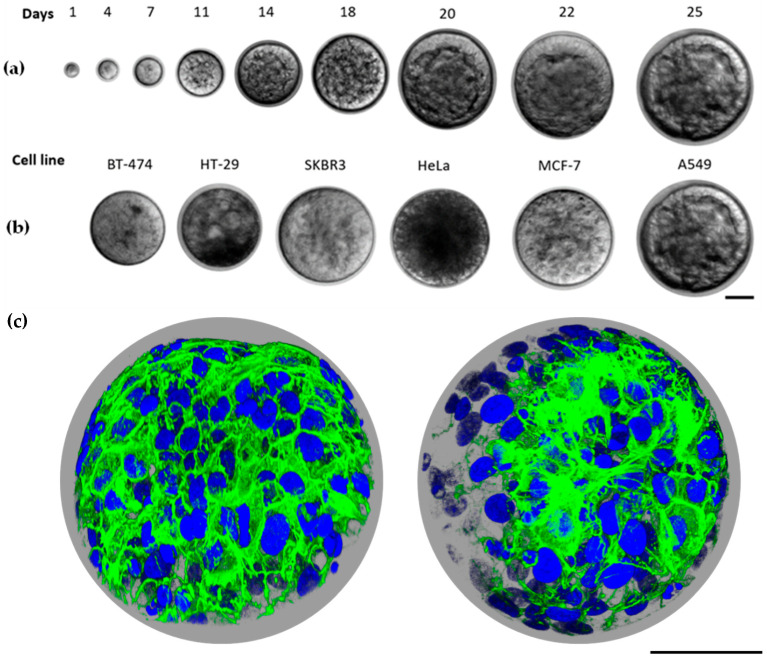
Tumor spheroids growth in the hydrogel: (**a**) Brightfield images of A549 cell line cells growing from single cells on Day 1 to tumor spheroids with an average size of around 150 µm on Day 25; (**b**) Grown tumor spheroids of BT-474, HT-29, SK-BR-3, HeLa, MCF-7 and A549 cell lines on Day 25, scale bar indicates 50 µm; (**c**) Confocal image of MCTSs, cells were stained with DAPI and Phalloidin, scale bar indicates 50 µm.

In order to demonstrate the potential of our alpha-acrylate hydrogel in ECM-mimicking applications, we utilize the hydrogel as a 3D scaffold for tumor spheroid formation. Employing hydrogels as a platform for 3D culture not only can offer cell suspension and nonadherent conditions for aggregation into MCTSs but also allows cancer stem cells (CSC) expansion in a controllable manner [19]. To explore how hydrogels accommodate and facilitate cell growth, we adjusted the mechanical properties of the hydrogels based on our prior research findings [28]. We discovered that softer gels, with a stiffness of around 1 kPa, promote cell expansion into tumor spheroids. Therefore, 3% of hydrogel was chosen. At this concentration, the gelation took place slowly, allowing the homogenous distribution of the cell suspension in the mixture to avoid aggregation and precipitation. After around 5 min, a relatively strong network formed, where the cells positions were fixed. Complete gelation was achieved in 30 min, then the cell medium was added. We chose 6 different cell lines, A549 (human lung cancer), BT-474 (ER/HER2-positive human breast cancer), MCF-7 (human breast cancer), HeLa (human cervical cancer), HT-29 (human colorectal cancer), and SK-BR-3 (HER2-positive human breast cancer), for encapsulation in the well-tuned hydrogel. The growth was monitored by using brightfield microscopy. The encapsulated cells were homogeneously distributed in the hydrogel and were nourished by cell medium (Figure S7). At first, single cells expanded into small spheroids with the support of the hydrogel’s structure. As the hydrogel swelled, the mesh size of the network increased, providing a highly flexible network environment for the spheroids to grow into larger tumor spheroids. Besides, due to the dynamic nature of the hydrogel, higher cell concentration in the later stages of the culture allowed cells to aggregate into substantially bigger tumor spheroids (Figure 5a,b). All cell lines successfully formed tumor spheroids after 3 weeks of culture. Notably, the growth patterns were similar across all the cell lines despite their distinct cell types and characteristics. In Figure 5c, a 3D scan of a stained tumor spheroid was imaged under a confocal microscope. Due to the limitation of the microscope, only the upper part of the tumor spheroid was presented. The sphere clearly demonstrated that the cells not only aggregated into clusters but formed spherical morphology, thereby developing into MCTSs.

**Figure 6:**
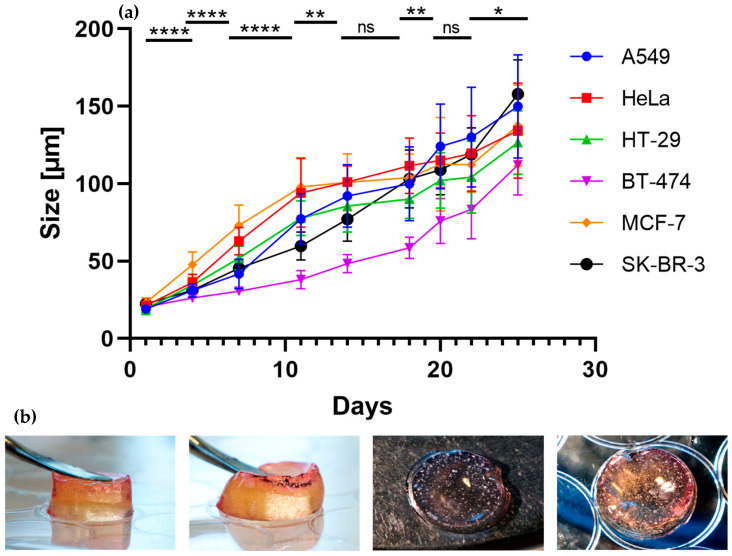
Size determination of tumor spheroids in hydrogel matrix; (**a**) Comparative size changes with all six different cell lines from within a culture period of 25 days. Data are presented as mean ± standard deviation, n = 25, *t*-test, shown for A549 cell line (see Table S1), * *p* < 0.05, ** *p* < 0.01, **** *p* < 0.0001, ns denotes no significance *p* > 0.05; (**b**) Photographs of tumoroids-encapsulated hydrogel after incubation in cell medium for 2 months. The numerous white dots inside the gel are grown MCTSs whose size is visible to the eyes.

The A549 cell line grew into tumor spheroids with an average size of 149.87 µm, with an average size of 134.19 µm for HeLa, 126.59 µm for HT-29, 112.07 µm for BT-474, 137.56 µm for MCF-7 and 157.86 µm for SK-BR-3 was observed after 25 days of incubation, respectively (Figure 6a). The microscopic images show the progression of the tumor spheroids from Day 1 to Day 25. All grown tumor spheroids are of similar shape with different sizes (Figure 5a,b). The difference could be attributed to the preferences of different cell lines in the tumor microenvironment (TME). The spheroid growth is influenced not only by the hydrogel’s stiffness but also by the compatibility of the mesh size with the rate of growth, which can impose constraints on expansion. Observations revealed that tumor spheroids tend to be larger with the size of 150–200 µm when positioned on the upper surface of the hydrogel, comparing to those at the bottom of the gel (~50 µm). This is because they benefit from improved access to oxygen and fresh cell medium. Furthermore, a relatively greater swollen hydrogel layer and increased network flexibility anticipated at the interface could facilitate tumoroids to grow bigger. MCTSs with relatively homogeneous size distributions can be observed in Figure S7. Nevertheless, the evidence demonstrates that alpha-acrylate hydrogel can serve as a universal platform for tumor spheroid’s growth while remaining structurally stable even after being immersed in a cell medium for a duration of two months (Figure 6b). This nondegradable nature ensures consistent stiffness over the desired timeframe, preserving the microenvironment necessary for specific cell lines. Unlike degradable hydrogels, which alter in stiffness through degradation, this characteristic raises the potential for a 3D culture of organoids, particularly considering the extended culture period required in contrast to tumoroids. It also holds promise for applications in tissue engineering and stem cell research.

## 3. Conclusions

In summary, the nondegradable hydrogel made from alpha-acrylate functionalized dPG and 4-arm PEG thiol at 3% *w*/*v* with 1 kPa stiff serves as an excellent supporting scaffold for various types of cancer cell lines such as A549, HeLa, HT-29, BT-474, MCF-7, and SK-BR-3. These cell lines are in situ encapsulated into a gel matrix and later form 3D tumor spheroids with standard culturing protocol and yet achieve an average tumoroid size at approximately 150 µm after 25 days of incubation with no observable toxicity. We believe that this gel platform can be a potential alternative not only for 3D tumoroid growth but also for organoid formation since the hydrogel has been proven to be stable for long time preservation.

## 4. Materials and Methods

### 4.1. Materials

All chemicals were purchased from Merck KGaA, Darmstadt, Germany and/or its affiliates and used without any further purification unless otherwise declared. Diethyl ether (100%) and DCM (100%) were purchased from VWR chemicals. N,N-Dimethylformamide (DMF) (99.8%), KOH (pellets), NaN_3_ (99%), MeOH (99.9%), THF (99.8%) and Chloroform were purchased from Thermo Fisher Scientific, Waltham, MA, USA. Ethyl 2-(bromomethyl)acrylate was purchased from TCI Deutschland GmbH (Eschborn, Germany). The average weight molecular weight of 10 kDa of dPG (Figure 1) was prepared as a previously reported procedure [34] in our group.

### 4.2. Synthesis of dPG alpha acrylate

The functionalization degree of alpha acrylate group onto dendritic polyglycerol (dPG) was calculated based on the number of theoretical free OH groups of the glycidol monomer. Ideally, a glycidol has 1 free OH group. So a 10 kDa dPG should contains approximately 135 groups of free OH (calculated by 10,000/74). If all 135 groups of free OH convert to a new functional group, it means this 10 kDa dPG polymer has 100% functionalization degree. Therefore, all the estimated equivalents of each reagent in the synthetic reaction are based on this calculation of the degree functionalization. 10 kDa dPG (1 eq., 0.1 mmol, 1 g, Figure S1) was dried in vacuum prior to being dissolved by DMF (10 mL). NaH (8 eq., 0.8 mmol, 0.019 g) was added to the solution which was then stirred at room temperature for 1 h. The reaction flask was later cooled down by an ice bath and Ethyl 2-(bromomethyl)acrylate (9 eq., 0.9 mmol, 0.124 mL) was added dropwise to the reaction mixture and it was run for 1 day. Afterward, the crude mixture was purified by dialysis with a 2 kDa cutoff dialysis tube in water for 2 days. After the purification, the pure product was stored as an aqueous solution and its concentration was calculated. Based on the concentration and total volume, the final yield was calculated and resulted in 83%. The functionalization degree of alpha acrylate was calculated by the area under a peak in the ^1^H NMR spectrum based on the NMR end-group analysis. It shows that there is 5% alpha acrylate decorated onto the dPG polymer (calculated by 0.05 × 100/1), which is roughly estimated to be 7 groups (calculated by 135 × 5%). ^1^H NMR (700 MHz, D_2_O, δ (ppm)): 0.92 (3H, broad s, initiator backbone), 1.36 (3H, broad s), 3.58–4.43 (m, backbone repeating units), 6.02–6.06 (1H, d), and 6.41 (1H, s) (Figure S2).

### 4.3. Synthesis of 4-arm PEG OMs

To a DCM (30 mL) solution of dried 10 kDa 4-arm polyethylene glycol (4-arm PEG, 1 eq., 0.3 mmol, 3 g, Figure S3)) was added an excess of Triethylamine (15 eq., 4.5 mmol, 0.63 mL). The reaction flask was then cooled down by an ice bath, followed by adding an excess of methanesulfonyl chloride (12 eq., 3.6 mmol, 0.28 mL). The reaction mixture was stirred for 1 day. The next day, the crude mixture was washed quickly with brine, later dried with Na_2_SO_4_ for 30 min and concentrated. The product in con-centrated DCM solution was then precipitated in cooled Ether and finally dried for 1 day in vacuum at room temperature. The final precipitated product is white with a 92% yield. 1H NMR (500 MHz, CDCl_3_, δ (ppm)): 3.08 (3H, s), 3.40–3.78 (m, polymer backbone), 4.36–4.38 (2H, t, J = 5 Hz) (Figure S4).

### 4.4. Synthesis of 4-arm PEG Thiol

4-arm PEG OMs (1 eq., 0.276 mmol, 2.76 g) was suspended in 1-propanol (30 mL), followed by the addition of an excess of thiourea (20 eq., 5.52 mmol, 0.42 g). The reac-tion mixture was then heated up at 80 °C which made all suspension become solution and it was stirred for 1 day. Next, the solvent was removed from the reaction flask and water (30 mL) was then added to dissolve the crude mixture, followed by the excessive addition of KOH (20 eq., 5.52 mmol, 0.31 g). The solution was then heated up and 80 °C and stirred for 1 day. Afterward, tris(2-carboxyethyl)phosphine hydrochloride (4 eq., 1.1 mmol, 0.32 g) was added to the reaction flask which was then run for 2 h at room temperature. Next, The purification took place. The crude product was first extracted quickly by DCM, then dried with Na_2_SO_4_ and concentrated with a rotary evaporator. The product in DCM solution was precipitated in cooled Ether and dried in vacuum for 1 day at room temperature. The resulting product was pale yellowish precipitated with 81% yield. 1H NMR (500 MHz, CDCl_3_, δ (ppm)): 1.57–1.60 (1H, t, J = 5 and 10 Hz), 2.67–2.70 (2H, quat, J = 5 Hz), 3.40–3.75 (m, polymer backbone) (Figure S5). The amount of thiol groups at the end chain of each arm was quantified by Ellman’s assay protocol from Thermo Fischer Scientific by using the standard calibration curve of cys-teine in different concentrations. The resulting number of thiol groups is approximate-ly 3.5 groups per 4-arm PEG molecule.

### 4.5. Rheological Experiment

The Rheological performance was conducted by Malvern Kinexus Prime Lab+. The hydrogel samples were mechanically characterized in triplication by using an 8-mm parallel plate at 37 °C with 0.05 N force and analyzed by oscillatory amplitude sweep and frequency sweep (0.1% strain) as well as viscosity (2 min ramp time) tests. The stiffness values of hydrogel samples were directly related to the shear storage modulus at 1 Hz. The mesh size was calculated from the stiffness or shear storage modulus at 1 Hz, by using the classical theory of rubber elasticity [35,36,37] as shown below:

$$r={\left(\frac{6RT}{\pi N_{Av}G}\right)}^{\frac{1}{3}}$$

where *r* is mesh size (nm), *R* is the gas constant (8.314 m^3^·Pa·K^−1^·mol^−1^), *T* is the temperature (K), π is Pi constant (3.142), *N_Av_* is Avogadro’s number (6.022 × 1023 mol^−1^) and *G* is shear storage modulus (Pa).

### 4.6. Cell Culture

All cell lines were obtained from DSMZ (German Collection of Microorganisms and Cell Cultures GmbH, Braunschweig, Germany), cultured in Dulbecco’s Modified Eagle Medium (DMEM, high glucose, GlutaMAX, Gibco), which was supplemented with 10% (*v*/*v*) fetal bovine serum (FBS, Gibco) and 1% (*v*/*v*) of penicillin-streptomycin solution (Gibco). Cells were cultured at 37 °C with 5% CO_2_ in a culture flask.

### 4.7. Hydrogelation for MCTSs Formation

To prepare 300 µL hydrogel at 3% *w*/*v* concentration for the MCTSs formation experiment, 10% *w*/*v* DMEM solution of 4-arm PEG thiol (0.7 µmol, 70 µL), 21.3% *w*/*v* dPG alpha acrylate aqueous solution (0.23 µmol, 11 µL), cell solution (estimated 10,000 cells, 50 µL) and 219 µL DMEM were simply mixed in a well of a 48-well plate. All cells were in situ encapsulated during gelation. The hydrogel sample was left in a cell incu-bator for 4 h prior to the addition of 400 µL DMEM on top of the gel for cell growth, fresh medium was changed every other day. The growth of MCTS was monitored under BrightField with Zeiss Axio Observer Z1 microscope (Jena, Germany).

### 4.8. MCTSs Staining and Imaging

The grown MCTSs were firstly fixed with 4% paraformaldehyde at room temper-ature for 30 min, then washed with DPBS 3 times. Then, DAPI and Phal-loidin-iFluoro594 reagent (Abcam, Cambridge, UK) were used to stain the cell nuclei and cytoskeletons for 30 min, followed by washing with DPBS 3 times. The stained MCTSs were carefully transferred from a 48-well plate to an 8-well Miccroscope µ-Slide (ibidi, Gräfelfing, Germany) and imaged by Leica SP8 confocal microscope (Wetzlar, Germany).

### 4.9. Statistical Analysis

All tests were conducted in at least three independent sessions. The quantified data are expressed as mean ± SD. GraphPad Prism was employed for statistical analysis. Differences between the tumoroid growth with time were analyzed using two sample unpaired *t*-test.

## Data Availability

The data presented in this study are openly available in article.

## Supplementary Materials

The following supporting information can be downloaded at: https://www.mdpi.com/article/10.3390/gels9120938/s1, Figure S1: 1H NMR (500 MHz, D_2_O, δ (ppm)) spectrum of 10 kDa dPG; Figure S2: 1H NMR (700 MHz, D_2_O, δ (ppm)) spectrum of 10 kDa dPG alpha acrylate; Figure S3: 1H NMR (500 MHz, CDCl_3_, δ (ppm)) spectrum of 10 kDa 4-arm polyethylene glycol (PEG); Figure S4: 1H NMR (500 MHz, CDCl_3_, δ (ppm)) spectrum of 10 kDa 4-arm PEG OMs; Figure S5: 1H NMR (500 MHz, CDCl_3_, δ (ppm)) spectrum of 10 kDa 4-arm PEG thiol; Figure S6: Shear viscosity graph of hydrogel sample at 37 °C at different incubation times; Table S1: *p*-value for the MCTS growth of each cell line; Figure S7: Brightfield images of cancer cell line A549, BT-474, HT-29, SK-BR-3, HeLa, and MCF-7 growing from single cells on Day 1 to tumor spheroids with an average size of around 150 µm on Day 25, scale bar indicates 100 µm.
