# Supplementary material for: Polyglycerol-Based Hydrogel as Versatile Support Matrix for 3D Multicellular Tumor Spheroid Formation
Source: Gels. 2023 Nov 29;9(12):938. doi: 10.3390/gels9120938 (PMC10742718; doi:10.3390/gels9120938)
Supplement: Supplementary file 1 [file gels-09-00938-s001.zip › gels-2723872-supplementary.pdf]

Supplementary materials

Polyglycerol-Based Hydrogel as Versatile Support Matrix for 3D Multicellular Tumor Spheroid Formation

Boonya Thongrom [1#], Peng Tang [2#], Smriti Arora* and Rainer Haag*

[1] b.thongrom@fu-berlin.de
[2] mauricetangp@zedat.fu-berlin.de
* Correspondence: smriti@zedat.fu-berlin.de and haag@zedat.fu-berlin.de
[#] These authors contributed equally to this work.

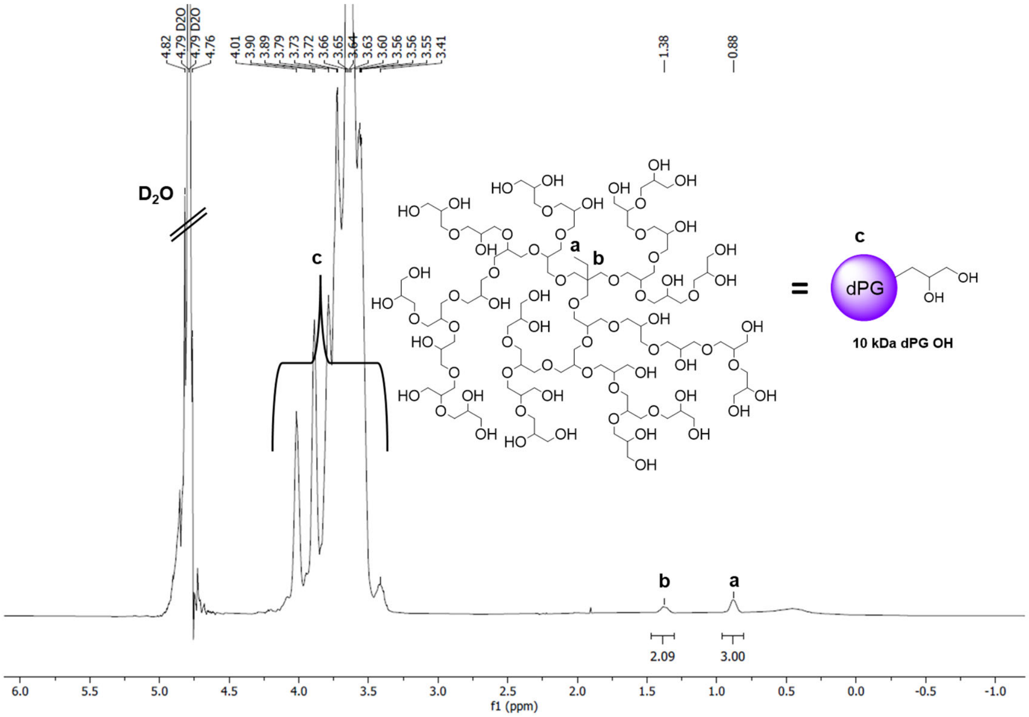

Figure S1. ^{1}H NMR (500 MHz, D_2O, δ (ppm)) spectrum of 10 kDa dPG.

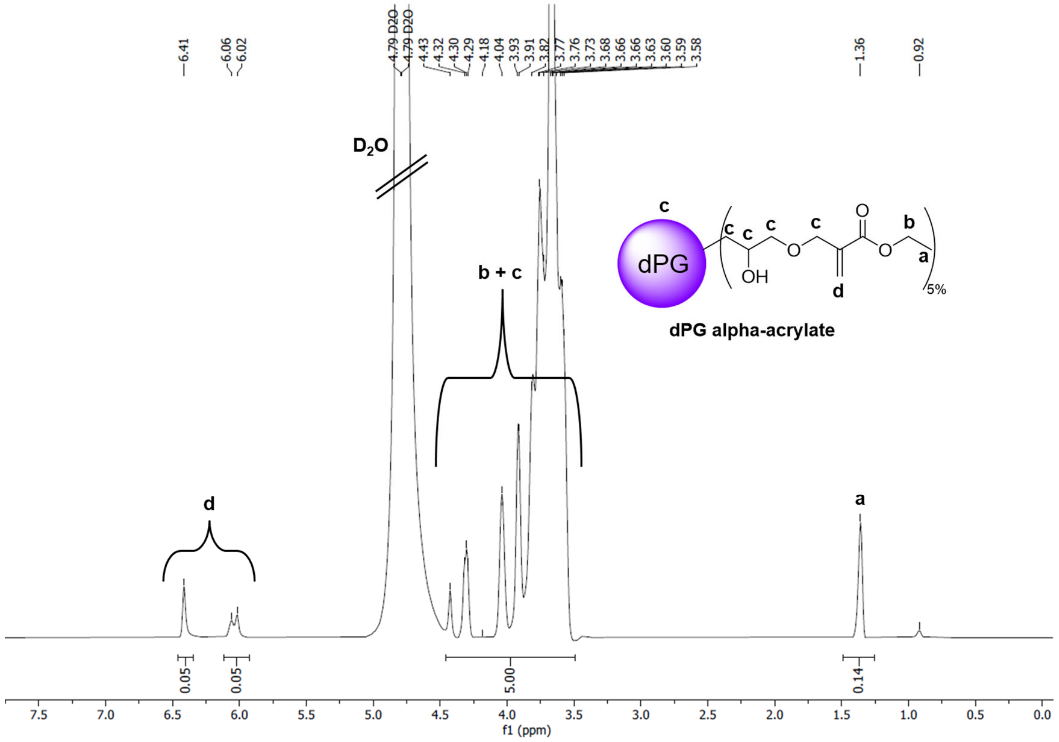

Figure S2. ^{1}H NMR (700 MHz, D_2O, δ (ppm)) spectrum of 10 kDa dPG alpha acrylate.

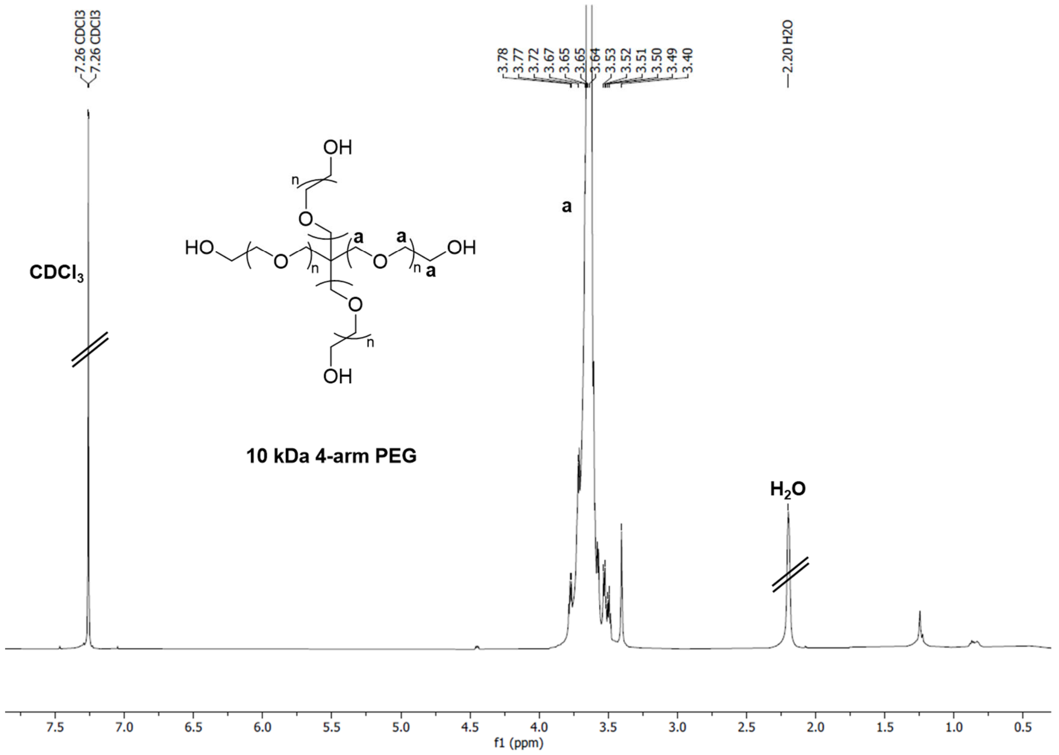

Figure S3. ^{1}H NMR (500 MHz, $CDCl_3$, δ (ppm)) spectrum of 10 kDa 4-arm polyethylene glycol (PEG).

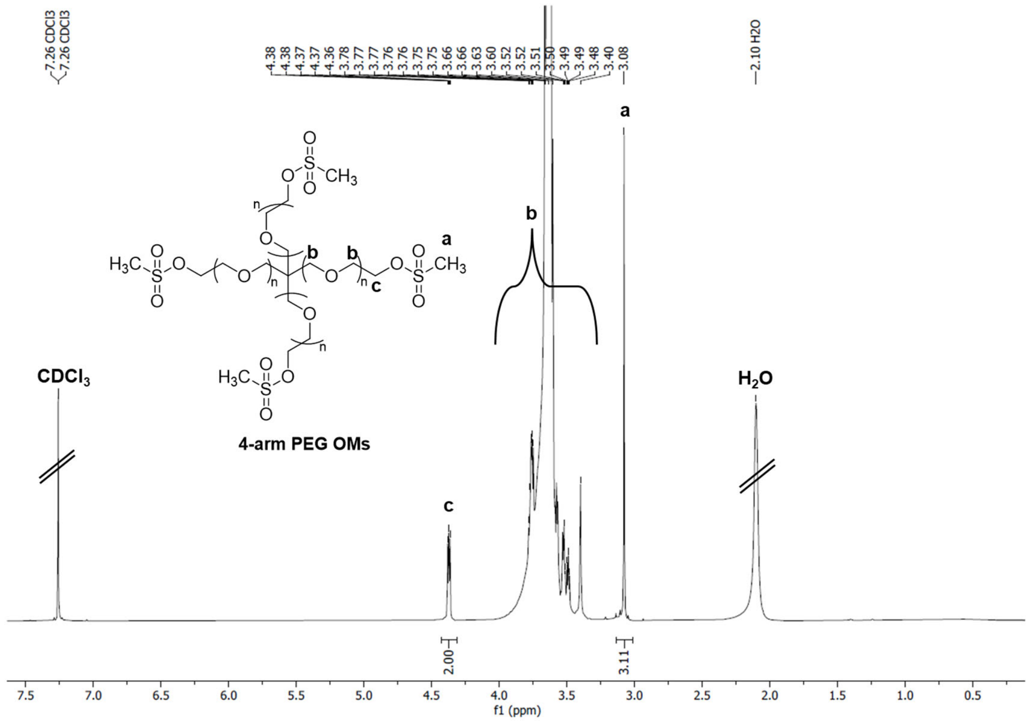

Figure S4. ^{1}H NMR (500 MHz, CDCl$_3$, δ (ppm)) spectrum of 10 kDa 4-arm PEG OMs.

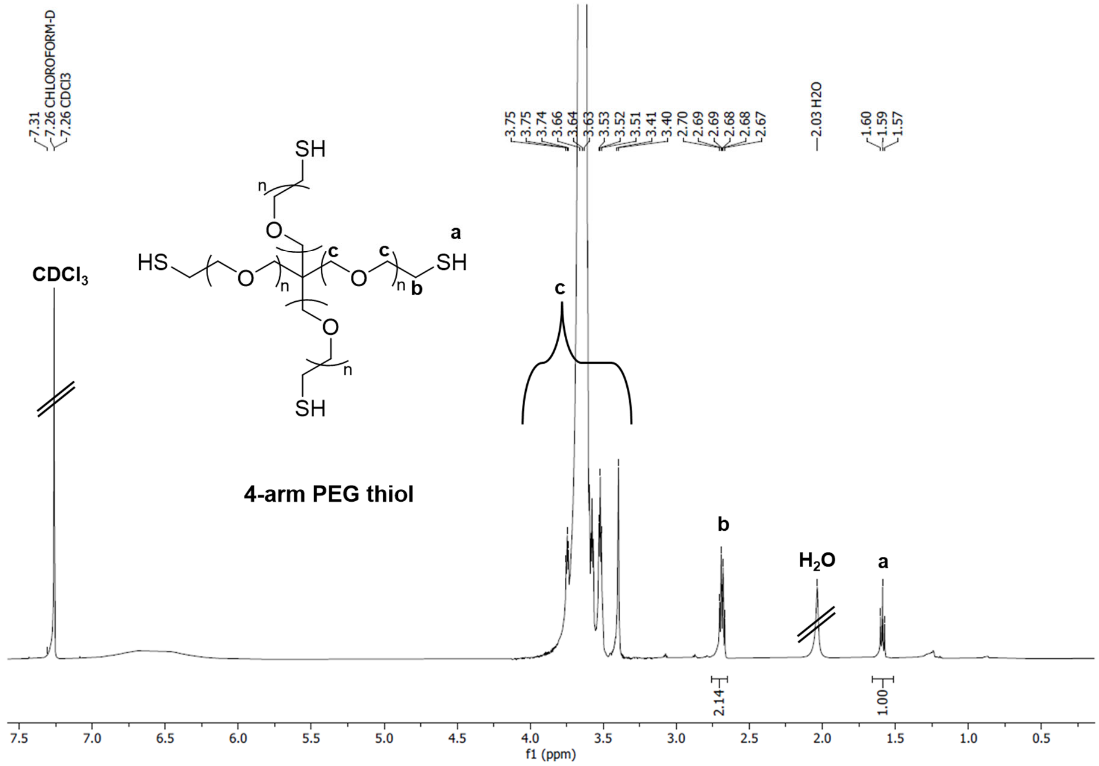

Figure S5. ^{1}H NMR (500 MHz, CDCl$_3$, δ (ppm)) spectrum of 10 kDa 4-arm PEG thiol.

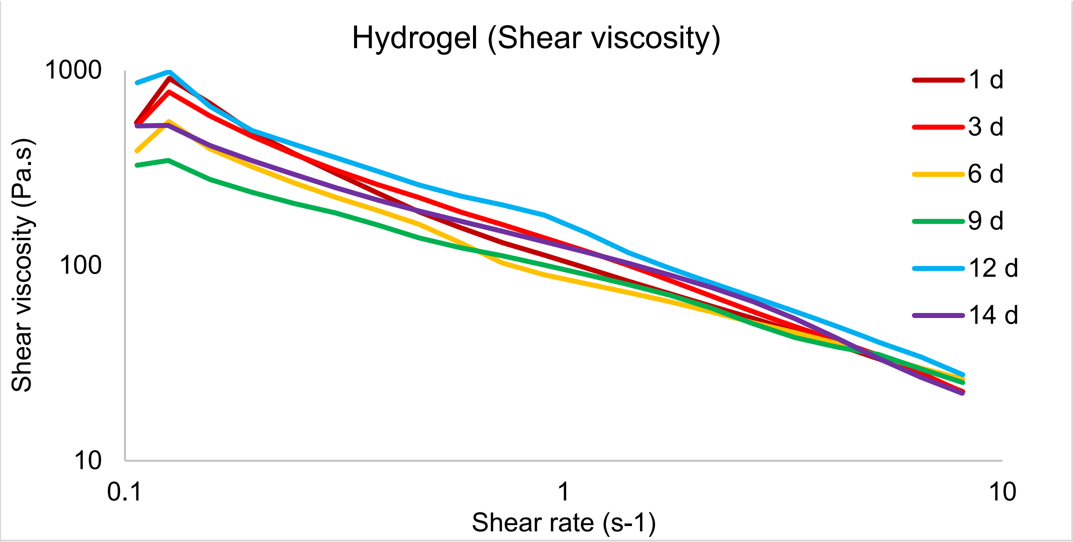

Figure S6. Shear viscosity graph of hydrogel sample at 37 °C at different incubation times.

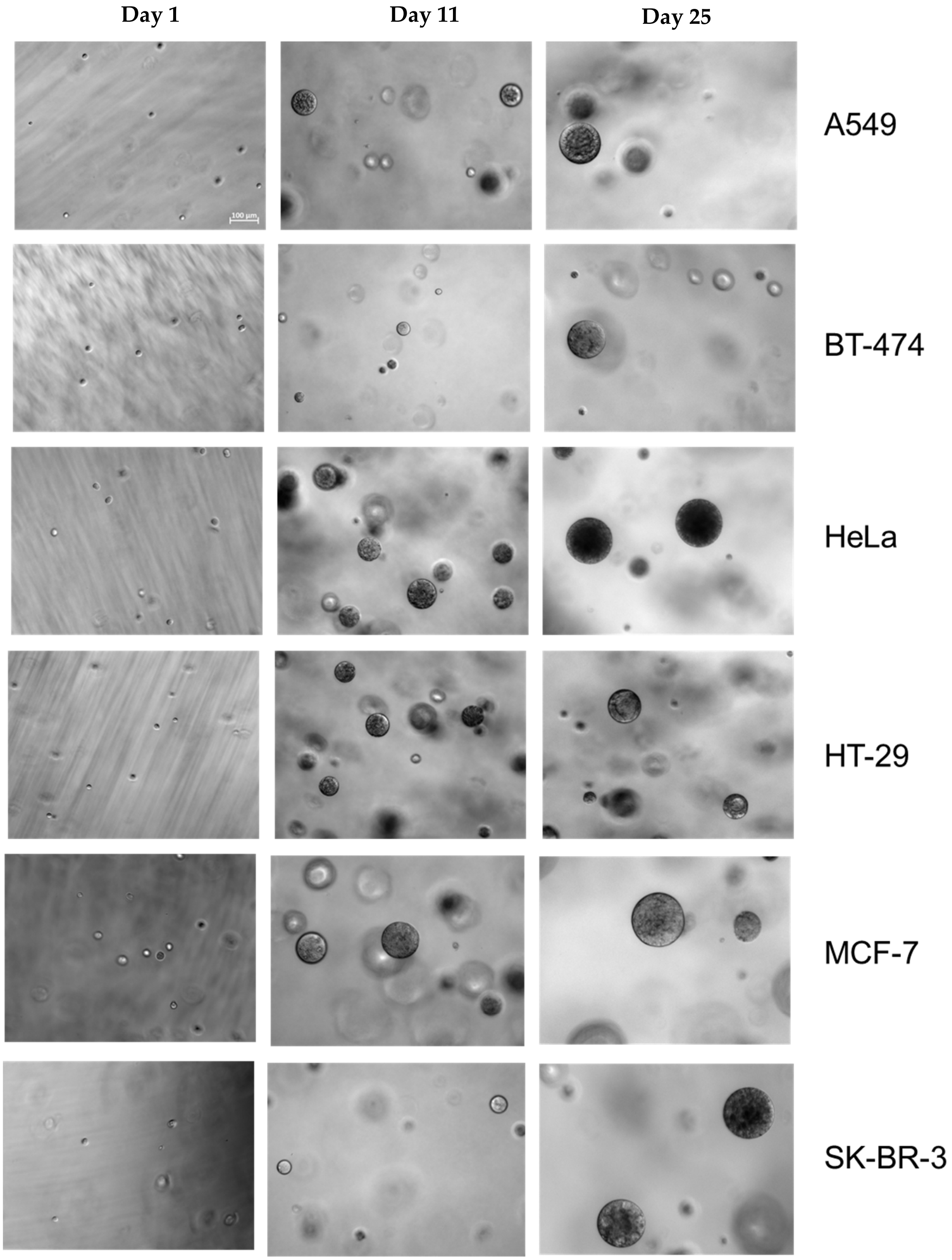

Figure S7. Brightfield images of cancer cell line A549, BT-474, HT-29, SK-BR-3, HeLa, and MCF-7 growing from single cells on day 1 to tumor spheroids with an average size of around 150 µm on day 25, scale bar indicates 100 µm.

Table S1. p-value for the MCTS growth of each cell line.

	D1 vs. D4	D4 vs. D7	D7 vs.D11	D11 vs. D14	D14. vs. D18	D18 vs. D20	D20 vs. D22	D22 vs. D25
**A549**	****	****	****	**	ns	**	ns	*
**HeLa**	****	****	****	ns	*	ns	ns	ns
**HT29**	****	****	****	ns	ns	**	ns	***
**BT474**	****	****	****	****	****	****	ns	****
**MCF7**	****	****	****	ns	ns	ns	ns	***
**SKBR3**	****	****	****	****	****	ns	*	****
